# ASSOCIATIONS OF VISUAL IMPAIRMENTS AND OCULAR DISEASE WITH BRAIN VOLUMES AND DEMENTIA IN UK BIOBANK

**DOI:** 10.1093/geroni/igad104.0633

**Published:** 2023-12-21

**Authors:** Erin Ferguson, Peter Buto, Jingxuan Wang Mary Thoma, Shea Andrews Kaitlin Casaletto, Tom Hoffmann Helene Choquet, Kristine Yaffe, M Maria Glymour, Willa Brenowitz

**Affiliations:** University of California, San Francisco, San Francisco, California, United States; University of California San francisco, San Francisco, California, United States; University of California San francisco, San Francisco, California, United States; University of California, San Francisco, California, United States; University of California San francisco, San Francisco, California, United States; UCSF, San Francisco, California, United States; University of California San Francisco, San Francisco, California, United States; Kaiser Permanente Center for Health Research, Portland, Oregon, United States

## Abstract

Visual impairments and ocular diseases are emerging potentially modifiable risk factors for dementia, however, causal relationships are poorly understood and vision impairment may also impact dementia evaluations. Few studies have examined associations of multiple measures of visual impairments with markers of neurodegeneration. We investigated associations of visual acuity and history of ocular diseases with dementia and brain MRI outcomes. UK Biobank participants aged 40-70 without dementia at assessment (n=496,938) self-reported history of cataract, glaucoma, age-related macular degeneration Subsets had visual acuity exams and refractive error measured by autorefractor (n=117,163) and brain MRIs (n=33,923). In cox proportional-hazard models adjusted for demographics and other health conditions, history of cataracts was associated with higher risk of all-cause dementia (HR:1.19: 95%CI: 1.09-1.31) but other eye conditions were not associated. History of cataract was associated with significantly smaller total gray matter volume (β=-2483 mm3, 95% CI: -4225 to -741) but not an AD brain signature based on cortical thickness. Worse than 20/40 visual acuity was associated with dementia (HR = 1.40, 95% CI: 1.11-1.77) but myopia defined based on refractive error was not, and neither were associated with total or regional volumes on brain MRI. History of cataract and binary 20/40 vision are associated with dementia risk. Our results suggest that cataract, but not myopia, may increase risk of neurodegeneration independent of typical AD, but more investigation is needed.

